# The Effects of *C. lacerata* on Insulin Resistance in Type 2 Diabetes Patients

**DOI:** 10.1155/2022/9537741

**Published:** 2022-02-22

**Authors:** Arim Choi, Jung Hye Kim, Hye-Kyung Chung, Chul Woo Ahn, Hee Joon Choi, Yu-Sik Kim, Ji Sun Nam

**Affiliations:** ^1^Division of Endocrinology, Department of Internal Medicine, Gangnam Severance Hospital, Yonsei University College of Medicine, Republic of Korea; ^2^Severance Institute for Vascular and Metabolic Research, Yonsei University College of Medicine, Republic of Korea; ^3^Gangnam Severance Hospital, Biochemical Research Center, Republic of Korea

## Abstract

**Background:**

Several experimental studies have suggested beneficial effects of *Ceriporia lacerata* on glucose metabolism. However, there has been no human study assessing the effects of *C. lacerata* on glucose metabolism. Therefore, we investigated whether *C. lacerata* improves glucose control and insulin resistance in type 2 diabetes patients.

**Methods:**

Ninety patients diagnosed with type 2 diabetes (T2DM) for more than 6 months were enrolled. Subjects were randomly divided into placebo (*n* = 45) or *C. lacerata* (*n* = 45) groups and then assigned to take placebo or *C. lacerata* capsules (500 mg/capsule) for a 12-week intervention period. Biochemical markers, including fasting glucose, 2-hour postprandial plasma glucose, and lipid profile levels, as well as insulin, c-peptide, and Hba1c, were measured. Furthermore, insulin sensitivity indices, such as HOMA-IR, HOMA-beta, and QUICKI, were assessed before and after the 12-week administration.

**Results:**

Eighty-four patients completed the study. There were no significant differences in fasting, postprandial glucose, HbA1c, or lipid parameters. HOMA-IR and QUICKI indices were improved at week 12 in the *C. lacerata* group, especially in subjects with HOMA-IR of 1.8 or more (*p* < 0.05). Fasting, postprandial c-peptide, and insulin levels decreased at week 12 in the *C. lacerata* group (*p* < 0.05). These significant differences were not observed in the placebo group.

**Conclusion:**

Twelve-week administration of *C. lacerata* in T2DM patients resulted in significant improvement in insulin resistance, especially in those with lower insulin sensitivity. A larger population study with a longer follow-up period and an effort to elucidate the mechanism is warranted to further assess the effects of *C. lacerata* on T2DM patients.

## 1. Introduction

Type 2 diabetes mellitus (T2DM), one of the most prevalent types of metabolic disorders worldwide, mainly results from impaired insulin sensitivity and defective insulin secretory function by pancreatic beta cells [1, 2]. Adequate amount of insulin and its action have to correctly meet the metabolic demand to regulate blood glucose level, and their defect leads to the pathogenesis of T2DM [3]. The rising prevalence of T2DM and its associated complications, including neuropathy, nephropathy, cardiopathy, and retinopathy, poses a major global health burden. Epidemiological data show alarming values that predict a dreadful future for T2DM. In most cases, hypoglycemic drugs, such as biguanides, thiazolidinediones, sulfonylureas, dipeptidyl peptidase-IV inhibitors, and sodium-glucose cotransporter 2 (SGLT2) inhibitors, are recommended for the management of hyperglycemia in patients with T2DM. However, many of the currently used hypoglycemic drugs are associated with various side effects, such as hypoglycemia, weight gain or loss, edema, and gastrointestinal distress, such as nausea, abdominal discomfort, and diarrhea [4]. Therefore, some patients are reluctant to take antidiabetic drugs and search for alternative ways, such as natural products or dietary supplements, to treat their diabetes.

Medicinal plants, a key player of all available therapies in phytomedicine, act as an alternative source of antidiabetic agents [5]. Antidiabetic bioactive compounds found from Korean medicinal plants so far include polysaccharides, terpenoids, flavonoids, sterols, and alkaloids.

According to past studies, mushrooms, especially polypore fungi, have been incorporated into the medicine of ancient civilizations worldwide. They exhibit nutraceutical potential from their antitumor, anti-inflammatory, anticoagulant, and hypotensive effects [6, 7]. Polypore species belonging to the genus *Ganoderma* are some of the oldest traditional medicines. In particular, many studies on *Ganoderma lucidum* extracts or isolates have underlined its anticancer, antiandrogen, immune-stimulating, antidiabetic, lipid-lowering, and anti-inflammatory activities [8, 9].

*Ceriporia lacerata* (CL, Aphyllophorales, Basidiomycota), which comes from white-rot fungus and is capable of degrading cellulose, hemicelluloses, and lignin in wood, have shown antidiabetic properties in high-fat-induced diabetic obese mice. In the study by Choi et al., *C. lacerata* showed antihyperglycemic effects by increasing glucose uptake in gastrocnemius muscles via adenosine monophosphate-activated protein kinase (AMPK) phosphorylation and increasing glucose transporter 4 (GLUT4) expression levels in mice [10]. However, there has been no clinical study to prove the beneficial effects of *C. lacerata* on glucose metabolism in type 2 diabetes patients.

Our study is aimed at investigating the effect of *C. lacerata* on improving glycemic markers, insulin resistance, and insulin sensitivity of T2DM patients through a 12-week randomized, double-blind, placebo-controlled clinical trial.

## 2. Methods

### 2.1. Subjects

Ninety patients aged 19 years or older, who were diagnosed with type 2 diabetes (HbA1c range from 7% to 9.9%) for more than 6 months and treated with oral antidiabetic agents at the time of enrollment, were pooled from Gangnam Severance Hospital. Subjects were to maintain their medications throughout the study. All of the participants were fully informed of the study protocol and expressed consent to participate. This study was approved by the Institutional Review Board of Gangnam Severance Hospital.

The following subjects were excluded from the study: prior diagnosis of chronic hepatic disease or elevated liver enzyme such as aspartate aminotransferase (AST) and alanine aminotransferase (ALT) over 3 times the upper normal range, diagnosis of impaired renal function (creatinine ≥ 3.5 mg/dL), corticosteroid therapy within the past 6 months, pregnant or lactating, and unable to fully understand and consent to the potential side effects of the study.

All participants were provided informed consent, and the study protocol was approved by the Institutional Review Board of Gangnam Severance Hospital (approval number: 3-2019-0185).

### 2.2. Test Materials

#### 2.2.1. Preparation of *C. lacerata*

*C. lacerata* mycelium was inoculated into the potato dextrose agar (Difco Co., Maryland, USA) medium and cultured at 25°C for 9 days. Liquid medium of *C. lacerata* mycelium was then mixed with 4 g/L of starch, 20 g/L of glucose, and 600 mL of purified water at 23°C for 10 days under pH 5 at a rotational speed of 300 rpm [11].

After the preculturing process, mycelium culture was transferred to liquid medium consisting of 12.5 g/L of sugar, 2.5 g/L of skim soybean meal, 2.5 g/L of starch, 0.125 g/L of antifoam, and 400 L of purified water. The medium was adjusted to pH 5 and incubated for 9 days at 23°C by injecting air (1.0 kgf/cm^2^) at a rotational speed of 100 rpm. The completed culture of *C. lacerata mycelium* was freeze-dried and pulverized and then used according to the capacity of each experimental group based on the dry weight. The source of *C. lacerata* was provided from 100% pure *C. lacerata mycelium* culture freeze-dried and pulverized.

#### 2.2.2. Clinical Sample Preparation

Magnesium stearate (SUN ACE, Singapore) was added to the submerged culture of *C. lacerata* mycelium and mixed for 20 minutes with a double-cone mix. The added amount of magnesium stearate was calculated as 1% of the total weight of *C. lacerata* mycelium. The test material was made into a tablet using a rotary tablet machine. Rectangular 30 PIN punch was used under 50 kN and 25 rpm to produce 505 mg tablets.

The tablets were uniformly coated with a coating machine. Then, 0.98% of hydroxypropyl methylcellulose (Lotte Chemical, Korea) was dissolved in alcohol to prepare a coating base. Placebo tablets used for clinical trials were prepared only by mixing lactose, crystalline cellulose, and caramel pigments.

The average weight of the test samples and placebo samples was 509 ± 4.38 mg and 511 ± 5.23 mg, respectively.

### 2.3. Design

Upon completion of the 4-week run-in period, the subjects were randomly assigned to the placebo group (45 subjects) and the *C. lacerata* mycelium group (45 subjects) for a 12-week intervention period. Subjects were randomly selected by using computer-generated random numbers. Group allocation was successfully double-blinded (for both investigators and participants) throughout the study.

During the 12-week intervention period, subjects were assigned to take two *C. lacerata mycelium* capsules (1,000 mg) after every meal which is equivalent to taking 3,000 mg of *C. lacerata* capsule per day for the experimental group and 3000 mg of placebo capsules for the placebo group per day.

#### 2.3.1. Anthropometric Parameters, Blood Pressure, and Biochemical Profiles

At baseline and after 12 weeks of intervention, subjects had their body weight and height measured with an electronic anthropometry measuring device (BSM370, BioSpace, Seoul, Korea) to the nearest 0.01 kg and 0.01 cm, respectively, under the light clothing after fasting of 8 hours. BMI was calculated as weight/height^2^ (kg/m^2^). Waist circumference (WC) was measured as the length midway between the lowest rib and iliac crest in the standing position using a circumference measuring tape (SECA200, SECA, Hamburg, Germany). Hip circumference was measured at the widest point of the hip to the nearest 0.1 cm. Blood pressure was measured by a digital sphygmomanometer (EASYX800, Jawon Medical, Seoul, Korea) after a 10-minute chair rest.

Blood and urine samples were taken at the same time after 8 hours of fasting. Simple urine test was analyzed for urine albumin/creatinine ratio (urine ACR). Blood samples were drawn from the antecubital vein and then centrifuged immediately (1,600 g, 10 minutes) and stored at -70°C until analysis. Fasting and 2-hour postprandial plasma glucose levels were checked using the oral glucose tolerance test (OGTT). In addition, fasting, postprandial c-peptide, and insulin levels were measured by collecting venous blood at 0 and 2 hours. Total cholesterol, high-density lipoprotein (HDL) cholesterol, low-density lipoprotein (LDL) cholesterol, and triglyceride levels were measured enzymatically using a chemical analyzer (Daiichi, Hitachi 747, Japan). Insulin, c-peptide, and Hba1c were measured using the high-performance liquid chromatography method. Serum blood urea nitrogen (BUN) and creatinine (Cr) levels were measured enzymatically using a chemical analyzer (AU5800, Beckman Coulter, Inc., Brea, CA, USA). Serum aspartate aminotransferase (AST), alanine aminotransferase (ALT), and gamma-glutamyl transferase (GGT) were measured for the liver function test (AU5800, Beckman Coulter, Inc., Brea, CA, USA). ACR was calculated as the random urine albumin divided by the random urine creatinine concentration. Estimated glomerular filtration rate (eGFR) was calculated using the CKD-EPI equation, which involves the serum creatinine value, gender, and age [12].

#### 2.3.2. Insulin Resistance

Insulin resistance was assessed by referencing the fasting glucose (mg/dL) and insulin levels (mcIU/mL) from homeostasis model assessment (HOMA-IR), HOMA-beta cell function (HOMA-beta) method, and quantitative insulin-sensitivity check index (QUICKI). HOMA-IR and HOMA-beta were calculated using the following formula [13, 14]: homeostasis model assessment of insulin resistance (HOMA‐IR) = [fasting glucose (mg/dL) × fasting insulin (mcIU/mL)/405] and HOMA‐beta cell function (HOMA‐beta) = 360 × fasting insulin (mcIU/mL)/(fasting glucose (mg/dL) − 63). QUICKI was also calculated from the following formula: QUICKI = 1/[log(I0) + log(G0)], where I0 is fasting insulin (mcIU/mL) and G0 is fasting glucose (mg/dL). Since QUICKI is the reciprocal of the log-transformed product of fasting glucose and insulin, QUICKI is a dimensionless index.

### 2.4. Outcome Measures

Primary outcomes were changes in the fasting plasma glucose, glucose tolerance test, and Hba1c at week 12. Secondary outcomes were insulin resistance indices and lipid profiles.

### 2.5. Monitoring of Compliance and Adverse Events

The compliance of patients was monitored once at 6 weeks of study by telephone contact and at the completion of the study. All subjects were instructed to keep taking the tablets and feel free to call whenever they have any symptoms or difficulty during the study. At the end of the study, participants were instructed to bring the tablets if they have any remaining drugs. For safety assessment, vital sign measurements (blood pressure, heart rate, and body temperature), blood and urine tests, and adverse event monitoring by questionnaire were conducted.

### 2.6. Statistical Analysis

Statistical analysis was performed using SPSS version 23.0 for Windows. All subject characteristics at baseline and all independent data were presented as mean ± SD. Within-group differences after intervention were analyzed by nonparametric paired Wilcoxon signed-rank test, and differences between groups postintervention were analyzed by using *t*-test of Mann-Whitney *U* test and Quade's rank analysis of covariates adjusted for initial value of each parameter. Analyses were declared statistically significant for *p* value < 0.05.

## 3. Results

### 3.1. Baseline Characteristics

**Figure 1:**
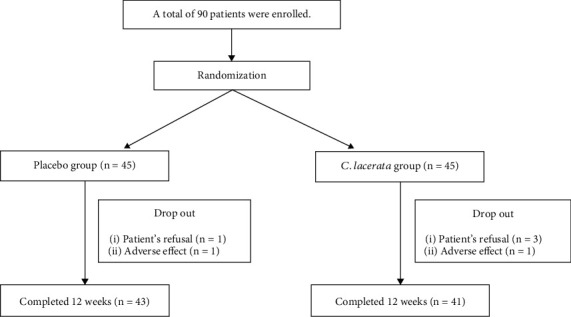
Flowchart of the clinical trial.

A total of 90 patients were enrolled in the study and randomized as the placebo (*n* = 45) and *C. lacerata* (*n* = 45) groups. During the study, we checked the compliance of patients by telephone and instructed all subjects to keep taking the tablets. Throughout this study, six patients dropped out (2 patients from the placebo group and 4 patients from the *C. lacerata* group) due to the lack of compliance or adverse effects. A total of 84 patient's (43 in the placebo group and 41 in the *C. lacerata* group) data were analyzed in the study (Figure 1). The mean age of study subjects was 61.9 years. The mean BMI was 25.4 kg/m^2^, and the mean diabetes duration was 10.7 years. Approximately one-third of the patients were taking a type of hypoglycemic agent, and two-thirds were taking two or more hypoglycemic agents. Nearly two-thirds of the patients were statin users. No significant differences were found in age, sex, BMI, DM duration, blood pressure, and fasting plasma glucose and Hba1c between the placebo and *C. lacerata* groups (Table 1).

### 3.2. Changes in Clinical Characteristics and Insulin Resistance Indices

No significant differences in the fasting plasma glucose, postprandial 2-hour glucose, and Hba1c were shown in both groups after 12 weeks of medication. Lipid profiles, such as the total cholesterol (TC), LDL cholesterol (LDL-C), HDL cholesterol (HDL-C), and triglyceride (TG), also exhibited no statistically significant changes after 12 weeks of drug administration. In the *C. lacerata* group, fasting, postprandial c-peptide, and insulin levels were significantly decreased after 12 weeks of drug administration. Although HOMA-beta and QUICKI showed no significant changes in both groups, HOMA-IR showed a tendency for improvement from 5.5 ± 5.19 to 4.3 ± 3.16, with a *p* value of 0.072. This change was not observed in the placebo group (Table 2).

### 3.3. Effects on Hepatic Markers and Renal Function

To investigate the hepatic and renal effects of *C. lacerata*, AST, ALT, and GGT levels were checked and liver fibroscan tests were performed. The ALT levels decreased from 29.3 ± 16.44 at baseline to 26.8 ± 13.94 after 12 weeks of *C. lacerata* therapy and showed statistical significance (*p* = 0.023). Furthermore, there was a significant intergroup difference in the amount of change in ALT level from baseline to week 12 between the *C. lacerata* and control groups. No significant changes in other hepatic markers and liver fibroscan measurements were observed in both groups. To check for the possibility of renal function effect on BUN, Cr, eGFR, cystatin C, and urine albumin, the urine Cr and urine ACR levels were compared; and no significant changes were found in any of the levels in both the placebo and *C. lacerata* groups (Table 3).

### 3.4. Subgroup Analysis

**Figure 2:**
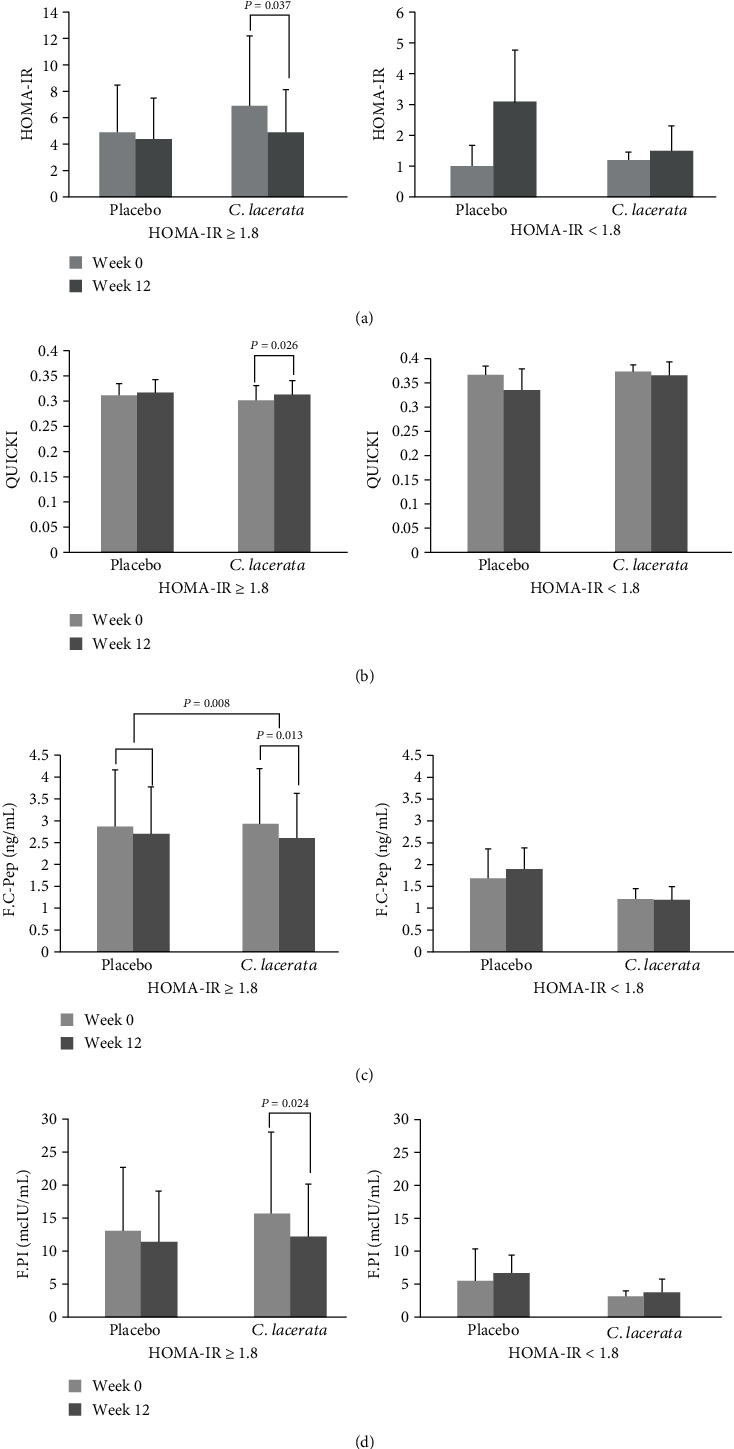
Subgroup analysis on the changes in (a) HOMA-IR, (b) QUICKI, (c) fasting c-peptide (F.C-pep), and (d) fasting plasma insulin (F.PI) between the placebo and *C. lacerata* groups after 12 weeks of intervention according to HOMA-IR.

Subjects were divided into 2 groups according to HOMA-IR. HOMA-IR of 1.8 was used as a cutoff point among various values for insulin resistance considering the characteristics of our study subjects [15]. The *C. lacerata* group with HOMA-IR of 1.8 or more showed statistically meaningful changes according to insulin resistance indices. HOMA-IR decreased and QUICKI increased, along with a decrease in fasting, postprandial C-peptide, and fasting insulin level (*p* < 0.05 versus baseline by Wilcoxon's signed-rank test). Such changes were not observed in the placebo group. The *C. lacerata* group with HOMA-IR less than 1.8 showed no statistically meaningful changes. Quade's rank analysis of covariates adjusted for initial values of each parameter was performed to estimate the statistical differences between groups. However, there was no significant difference among the placebo and *C. lacerata* groups (Figure 2, Table 4).

### 3.5. Adverse Effects

No serious adverse effects were found in either the *C. lacerata* group or the placebo group. One patient in the *C. lacerata* group dropped out due to constipation and deterioration of blood glucose. Within the placebo group, one patient dropped out due to constipation. However, no correlation was found between the symptoms and drug treatment. Hypoglycemia did not occur during the study period in both groups.

## 4. Discussion

The current study investigated the effects of *C. lacerata* on fasting glucose, Hba1c, insulin resistance indices, and lipid profile on patients with type 2 diabetes. The administration of *C. lacerata* for 12 weeks did not affect patients' fasting blood glucose, postprandial glucose, and Hba1c or lipid parameters. Regarding insulin resistance, fasting, postprandial insulin, and c-peptide significantly decreased, while HOMA-IR improved with trends toward significance (*p* = 0.072) from 5.5 ± 5.19 to 4.3 ± 3.16. However, in the subgroup analysis with patients whose HOMA-IR were 1.8 or above, HOMA-IR and QUICKI significantly improved from 6.9 ± 5.29 to 4.9 ± 3.24 (*p* = 0.037) and from 0.302 ± 0.028 to 0.313 ± 0.028 (*p* = 0.026), respectively. These findings suggest that *C. lacerata* is likely to have beneficial effects on reduction of insulin resistance in type 2 diabetes patients, especially in subjects with lower insulin sensitivity.

Polypore fungi have been used in various ways as food, commodities, and traditional medicine by different civilizations worldwide. In the past few decades, many mushrooms have been used as a key source for bioactive compounds, therapeutic supplements, and health-promoting food supplements. Growing efforts have been put into research to find out the medically active primary and secondary metabolites derived from polypores. The bioactive metabolites isolated from polypore species include triterpenoids, organic acids, flavonoids, coumarins, benzofurans, and N-containing compounds. These bioactive polypore extracts show abundant bioactive properties, such as antidiabetic, anti-inflammatory, anticancer, anticytotoxic, antimicrobial, and antioxidant activities [16].

In consideration of the known biological effects of fungal polypores, it appeared to us that *C. lacerata* extracted from white-rot fungus could have an antihyperglycemic role in T2DM patients. A previous observational study investigated the effects of *C. lacerata* (FBS0P) culture on diabetic mice by orally administrating FBS0P (500 mg/kg/d) to high-fat-fed diabetic mice. The FBS0P-treated mice showed improved insulin sensitivity in TNF-*α*-treated myotubes, as well as reduced serum insulin and c-peptide levels compared to the control, high-fat diet group. The related study also concluded that FBS0P-treated mice displayed increased amount of adenosine monophosphate-activated protein kinase (AMPK) phosphorylation and glucose transporter 4 (GLUT4) expression levels in gastrocnemius muscles by inducing GLUT4 translocation in C2C12 myotubes [10]. It is suggested that *C. lacerata* exhibits antihyperglycemic efficacy by improving insulin sensitivity in both a cell culture system and a high-fat-fed diabetic mouse model. While existing studies on *C. lacerata* are cell- and animal-based, studies based on type 2 diabetes patients are lacking so far [7, 17, 18]. These findings have led us to investigate the antihyperglycemic function of *C. lacerata* in humans diagnosed with T2DM. Our study showed a significant decrease in the level of fasting, postprandial c-peptide, and insulin levels in the *C. lacerata* group. Also, subgroup analysis with patients who had higher insulin resistance (HOMA-IR 1.8 or above) showed significant improvements in HOMA-IR and QUICKI, from 6.9 ± 5.29 to 4.9 ± 3.24 (*p* = 0.037) and from 0.302 ± 0.028 to 0.313 ± 0.028 (*p* = 0.026), respectively. These findings suggest the enhancement of insulin sensitivity in T2DM patients.

Insulin resistance, a reduced response of cells to the action of insulin, is a major finding in metabolic disorders such as diabetes mellitus type 2 (T2DM). Insulin resistance is not only related to hyperglycemia but also an important risk factor for cardiovascular and cerebrovascular diseases, various cancers, and neurodegenerative diseases [19, 20]. There are efforts to identify subjects with insulin resistance using HOMA-IR for preventive interventions in people with and without diabetes. Population-based studies for defining cutoff values of HOMA-IR in different geographic areas show that there is a great variability in the threshold of HOMA-IR levels to define insulin resistance. There are various cutoff values of HOMA-IR from 1.8 to 2.3 according to race/ethnicity, gender, age, with and without diabetes, and whether one takes antidiabetic medications [15]. Taking these into account, subjects were divided into subgroups according to their HOMA-IR level with a cutoff value of 1.8.

Improvement in insulin resistance has been reported to be related to chronic inflammation which can be measured by c-reactive protein, interleukin-6, or TNF-alpha [21] or oxidative stress [22]. Based on this fact, we have conducted correlational analysis between changes in CRP, advance glycation end product (AGE), and HOMA-IR. There were no differences in CRP and AGE before and after 12 weeks, and there were no significant correlations between changes in CRP and AGE with changes in HOMA-IR. Further studies are warranted to elucidate the mechanism of *C. lacerata* on improving insulin sensitivity.

In contrast to beneficial effects of *C. lacerata* on insulin sensitivity, it had no effects on blood glucose level and HbA1c. Possible reasons are relatively short duration of study and small number of study subjects. Also, there might have been some improvement if a more detailed test like continuous glucose monitoring or glucose tolerance test was conducted. For example, a study by Brasnyó et al. [23] has demonstrated that oral resveratrol improves insulin sensitivity assessed by HOMA-IR but not fasting or conventional postprandial glucose. Instead, there were significant improvements in time to maximum glucose and glucose levels at 25-35 minutes after test meal. There may be a possible direct inhibitory effect of *C. lacerata* on the pancreas since insulin and c-peptide levels were all decreased without concomitant improvement in blood glucose level. However, there was no deterioration in beta cell secretory function assessed HOMA-beta, and similar results have been demonstrated in similar studies conducted with natural products or complements like probiotics also show inconsistent results in terms of plasma glucose, HbA1c, and insulin [24], and mechanisms are also mostly not clearly known.

In terms of safety, there were serious adverse events including hepatotoxicity, nephrotoxicity, or hypoglycemia in the *C. lacerata* group. The ALT level was significantly lowered in the experimental group after 12 weeks of *C. lacerata*, and there was a significant intergroup difference in the changes in ALT level between the experimental and control groups. Nonalcoholic fatty liver disease is considered the hepatic manifestation of insulin resistance [25]. Chronic inflammation and mediators released from immune cells and adipocytes have been suggested to be mediators of liver damage in insulin resistance [26]. Thus, the improved insulin sensitivity may have attributed to such a change.

Our study has the strength of being the first human-based double-blinded study that investigated the clinical role of *C. lacerata* in patients diagnosed with T2DM. However, further studies on the activities and action mechanisms of *C. lacerata* improving the insulin sensitivity in humans are needed. Also, the issues of optimal dosage, bioavailability, and synergisms should be taken into account, since most of the previous studies were *in vitro* and animal-based and human-based clinical studies are still insufficient.

Other limitations of the current study are as follows. First, the number of human study subjects was too small to perform additional subgroup analyses. Second, the study duration was relatively short to assess the changes in glycemic parameters. However, this study was still valuable in that it was the first to investigate the clinical effects of *C. lacerata* on type 2 diabetes patients taking hypoglycemic medications. Third, all patients took the same dose of *C. lacerata*, regardless of their body weight and BMI. This study would have been more precise had the *C. lacerata* dose been titrated to each individual body weight or BMI. Lastly, subjects with hepatic or renal dysfunction were excluded from the study, which could make it difficult to generalize the effects and safety of *C. lacerata*.

## 5. Conclusions

To our knowledge, this is the first human-based study to investigate the clinical effects of *C. lacerata* on type 2 diabetes patients who are already on hypoglycemic medication. In this study, although *C. lacerata* did not show a glucose-lowering effect, it effectively improved insulin sensitivity especially in T2DM patients with lower insulin sensitivity possibly via its action in the skeletal muscle. Future investigations should be directed towards the mechanism and bioavailability of *C. lacerata* and to find out the antioxidant, anticytotoxic, and anti-inflammatory effects of *C. lacerata* in humans.

## Figures and Tables

**Table 1:** Subject characteristics at baseline.

	Total (*n* = 84)	Placebo group (*n* = 43)		*C. lacerata* group (*n* = 41)	*p* value
Gender (M/F)	57/27	28/15		29/12	NS^b^
Age (yrs)	61.9 ± 10.12	65.2 ± 8.77		58.5 ± 10.38	NS^a^
BMI (kg/m^2^)	25.4 ± 4.23	25.7 ± 4.21		25.1 ± 4.26	NS
DM durations (yrs)	10.7 ± 7.54	12.0 ± 8.35		9.3 ± 6.37	NS
DM medication					
Monotherapy	26 (30.9%)	9 (20.9%)		17 (41.5%)	NS^b^
Dual therapy	21 (25%)	12 (27.9%)		9 (22.0%)	
≥Triple therapy	37 (44%)	22 (51.2%)		15 (36.6%)	
Statin medication					
User	56 (66.7%)	29 (67.4%)		27 (65.9%)	NS^b^
Nonuser	28 (33.3%)	14 (32.6%)		14 (34.1%)	
SBP (mmHg)	127.9 ± 15.70	127.5 ± 15.86		128.4 ± 15.62	NS^a^
DBP (mmHg)	77.7 ± 11.52	76.1 ± 11.29		79.4 ± 11.60	NS
FPG (mg/dL)	165.7 ± 38.15	164.4 ± 44.16		171.7 ± 28.76	NS
Hba1c (%)	8.1 ± 0.93	8.1 ± 0.80		8.0 ± 0.76	NS

Data are presented as mean ± standard deviation (SD) or as numbers (%). ^a^Analyzed by independent *t*-tests and the *p* value represents the comparison to the placebo group. ^b^Analyzed by chi-square. BMI: body mass index; SBP: systolic blood pressure; DBP: diastolic blood pressure; FPG: fasting plasma glucose.

**Table 2:** Clinical characteristics of the study population.

	Placebo group (*n* = 43)			*C. lacerata* group (*n* = 41)			*p* value^b^
	Week 0	Week 12	*p* value^a^	Week 0	Week 12	*p* value^a^	
FPG (mg/dL)	164.4 ± 44.16	163.4 ± 45.26	0.477	171.7 ± 28.76	173.8 ± 38.82	0.765	0.352
2 hr Glc (mg/dL)	334.3 ± 72.33	341.3 ± 86.5	0.375	354.0 ± 65.86	357.6 ± 68.0	0.712	0.898
Hba1c (%)	8.1 ± 0.80	8.3 ± 1.13	0.047	8.0 ± 0.76	8.3 ± 1.06	0.016	0.287
F.C-pep (ng/mL)	2.7 ± 1.28	2.5 ± 1.04	0.689	2.5 ± 1.34	2.3 ± 1.09	0.001^∗^	0.440
P.C-pep (ng/mL)	6.0 ± 1.97	5.8 ± 1.9	0.764	5.5 ± 2.1	5.0 ± 1.86	0.001^∗^	0.136
F.PI (mcIU/mL)	11.6 ± 9.4	10.5 ± 7.24	0.336	12.9 ± 12.0	10.3 ± 7.84	0.014^∗^	0.347
P.PI (mcIU/mL)	34.8 ± 22.67	34.9 ± 22.35	0.678	36.4 ± 31.7	27.9 ± 21.52	0.009^∗^	0.109
HOMA-IR	4.3 ± 3.52	4.2 ± 0.03	0.432	5.5 ± 5.19	4.3 ± 3.16	0.072	0.199
HOMA-beta	46.1 ± 44.47	44.1 ± 34.55	0.957	45.6 ± 43.91	38.3 ± 34.81	0.089	0.593
QUICKI	0.3 ± 0.03	0.3 ± 0.03	0.541	0.3 ± 0.04	0.3 ± 0.04	0.287	0.741
TC (mg/dL)	164.6 ± 37.76	162.2 ± 36.5	0.184	172.6 ± 33.19	174.2 ± 40.10	0.401	0.300
LDL-C (mg/dL)	95.5 ± 28.27	92.8 ± 30.24	0.53	99.36 ± 24.74	101.4 ± 29.46	0.229	0.202
HDL-C (mg/dL)	46.7 ± 10.12	45.0 ± 10.33	0.027	49.7 ± 11.57	50.0 ± 12.32	0.711	0.523
TG (mg/dL)	153.0 ± 77.7	161.8 ± 75.3	0.149	160 ± 110.25	150 ± 90.0	0.166	0.220

Data are presented as mean ± standard deviation (SD). ^a^Nonparametric paired Wilcoxon signed-rank test was used to compare baseline data to those at 12 weeks in each group. ^b^Mann-Whitney *U* test was employed for analysis of difference between two groups. Statistically significant values are indicated ^a^within-group *p* value < 0.05 and ^b^between-group *p* value < 0.05. FPG: fasting plasma glucose; 2 hr Glc: 2-hour plasma glucose; F.C-pep: fasting C-peptide; P.C-pep: postprandial C-peptide; F.PI: fasting plasma insulin; P.PI: postprandial plasma insulin; HOMA-IR: homeostasis model assessment for insulin resistance; QUICKI: quantitative insulin-sensitivity check index; TC: total cholesterol; LDL-C: LDL cholesterol; HDL-C: HDL cholesterol; TG: triglyceride; NS: nonsignificant.

**Table 3:** Biochemical measurements of hepatic and renal function.

	Placebo group (*n* = 43)			*C. lacerata* group (*n* = 41)			*p* value^b^
	Week 0	Week 12	*p* value^a^	Week 0	Week 12	*p* value^a^	
ALT (IU/L)	25.3 ± 14.92	27.9 ± 22.2	0.741	29.3 ± 16.44	26.8 ± 13.94	0.023^∗^	0.018^∗^
AST (IU/L)	25.1 ± 10.4	26.8 ± 16.5	0.867	29.6 ± 17.56	29.0 ± 16.75	0.672	0.457
GGT (IU/L)	34.7 ± 31.52	36.9 ± 38.62	0.716	33.1 ± 25.41	31.4 ± 20.34	0.25	0.175
Liver fibroscan							
LSM score (kPa)	6.03 ± 3.006	6.28 ± 3.113	0.124	5.67 ± 2.427	5.94 ± 2.953	0.079	0.978
CAP score (dB/m)	265.3 ± 50.28	264.4 ± 51.96	0.867	267.6 ± 59.43	265.9 ± 53.79	0.919	0.905
BUN (mg/dL)	16.7 ± 4.5	16.8 ± 6.1	0.92	15.5 ± 5.71	15.3 ± 4.18	0.81	0.690
Cr (mg/dL)	0.8 ± 0.27	0.8 ± 0.25	0.872	0.8 ± 0.25	0.8 ± 0.21	0.269	0.826
eGFR (mL/min)	91.8 ± 24.94	92.6 ± 24.78	0.512	99.0 ± 22.86	97.8 ± 22.62	0.096	0.301
Cystatin C (mg/L)	0.9 ± 0.28	0.9 ± 0.3	0.063	0.9 ± 0.19	0.8 ± 0.19	0.202	0.933
Urine Alb (mg/dL)	6.9 ± 14.57	5.3 ± 8.1	0.988	10.0 ± 38.28	11.7 ± 43.15	0.769	0.101
Urine Cr (mg/dL)	125.5 ± 86.8	118.4 ± 64.77	0.885	127.7 ± 68.5	124.3 ± 67.18	0.987	0.754
Urine ACR (mg/gCr)	69.4 ± 160.74	54.1 ± 94.5	0.871	117.3 ± 423.9	115.0 ± 393.19	0.696	0.581

Data are presented as mean ± standard deviation (SD). ^a^Nonparametric paired Wilcoxon signed-rank test was used to compare baseline data to those at 12 weeks in each group. ^b^Mann-Whitney *U* test was employed for analysis of difference between two groups. Statistically significant values are indicated ^a^within-group *p* value < 0.05 and ^b^between-group *p* value < 0.05. ALT: alanine aminotransferase; AST: aspartate aminotransferase; GGT: gamma-glutamyl transferase; LSM: liver stiffness measurement; CAP: controlled attenuated parameter; BUN: blood urea nitrogen; Cr: creatinine; eGFR: estimated glomerular filtration rate; urine Alb: urine albumin; urine Cr: urine creatinine; urine ACR: urine albumin to creatinine ratio; NS: nonsignificant.

**(a):** 

	HOMA‐IR ≥ 1.8						*p* value^c^
	Placebo group (*n* = 35)			*C. lacerata* group (*n* = 32)			
	Week 0	Week 12	*p* value^a^	Week 0	Week 12	*p* value^a^	
Hba1c (%)	8.2 ± 0.79	8.3 ± 1.06	0.349	8.1 ± 0.79	8.3 ± 1.13	0.064	0.108
HOMA-IR	4.9 ± 3.56	4.4 ± 3.10	0.112	6.9 ± 5.29	4.9 ± 3.24	0.037^∗^	0.459
HOMA-beta	50.85 ± 46.50	48.39 ± 36.40	0.555	45.64 ± 54.84	43.89 ± 37.12	0.477	0.325
QUICKI	0.312 ± 0.023	0.317 ± 0.025	0.124	0.302 ± 0.028	0.313 ± 0.028	0.026^∗^	0.40
FPG (mg/dL)	167.1 ± 45.81	158.2 ± 36.9	0.127	176.7 ± 30.75	175.4 ± 40.21	0.721	0.186
2 hr Glc (mg/dL)	338.9 ± 65.76	158.2 ± 36.98	0.825	350.3 ± 64.58	353.1 ± 60.46	0.695	0.395
F.C-pep (ng/mL)	2.882 ± 1.286	2.698 ± 1.084	0.266	2.920 ± 1.280	2.610 ± 1.026	0.013^∗^	0.008^∗^
P.C-Pep (ng/mL)	6.293 ± 1.846	6.204 ± 1.845	0.864	6.113 ± 1.977	5.496 ± 1.754	0.025^∗^	0.185
F.PI (mcIU/mL)	13.02 ± 9.65	11.36 ± 7.67	0.094	15.65 ± 12.34	12.14 ± 7.98	0.024^∗^	0.202
P.PI (mcIU/mL)	37.63 ± 22.38	38.09 ± 22.94	0.701	43.20 ± 33.14	32.90 ± 22.31	0.107	0.289

**(b):** 

	HOMA‐IR < 1.8						*p* value^c^
	Placebo group (*n* = 8)			*C. lacerata* group (*n* = 9)			
	Week 0	Week 12	*p* value^a^	Week 0	Week 12	*p* value^a^	
Hba1c (%)	8.1 ± 0.87	8.7 ± 1.41	0.011	7.8 ± 0.62	7.9 ± 0.73	0.944	0.325
HOMA-IR	1.0 ± 0.69	3.1 ± 1.69	0.025	1.2 ± 0.26	1.5 ± 0.81	0.123	0.982
HOMA-beta	19.36 ± 11.99	25.27 ± 15.33	0.173	12.63 ± 5.03	14.37 ± 7.73	0.374	0.279
QUICKI	0.368 ± 0.017	0.336 ± 0.043	0.116	0.374 ± 0.014	0.366 ± 0.028	0.213	0.743
FPG (mg/dL)	148.8 ± 31.87	186.3 ± 70.11	0.249	155.8 ± 13.78	168.1 ± 36.32	0.263	0.155
2 hr Glc (mg/dL)	314.1 ± 99.07	357.9 ± 132.78	0.161	367.1 ± 74.46	372.7 ± 93.49	0.953	0.740
F.C-pep (ng/mL)	1.662 ± 0.689	1.885 ± 0.492	0.123	1.194 ± 0.245	1.178 ± 0.303	0.767	0.158
P.C-pep (ng/mL)	4.600 ± 2.001	4.246 ± 1.271	0.889	3.579 ± 1.226	3.271 ± 1.142	0.314	0.149
F.PI (mcIU/mL)	5.46 ± 4.89	6.60 ± 2.82	0.233	3.12 ± 0.84	3.76 ± 1.73	0.212	0.518
P.PI (mcIU/mL)	22.85 ± 21.14	20.69 ± 12.58	0.575	12.77 ± 6.26	11.42 ± 5.07	0.441	0.094

Data are presented as mean ± standard deviation (SD). ^a^Nonparametric paired Wilcoxon signed-rank test was used to compare baseline data to those at 12 weeks in each group. ^c^Quade's rank analysis of covariates adjusted for each glycemic marker initial values was performed to estimate the statistical differences between the placebo and *C. lacerata* groups' changes in glycemic markers after the 12-week intervention. Statistically significant values are indicated ^a^within-group *p* value < 0.05 and ^c^between-group *p* value < 0.05. HOMA-IR: homeostasis model assessment for insulin resistance; QUICKI: quantitative insulin-sensitivity check index; FPG: fasting plasma glucose; 2 hr Glc: 2-hour plasma glucose; F.C-pep: fasting C-peptide; P.C-pep: postprandial C-peptide; F.PI: fasting plasma insulin; P.PI: postprandial plasma insulin.

## Data Availability

Data generated or analyzed during this study are included in this published article. The data generated or analyzed during this study are available from the corresponding author upon request.
